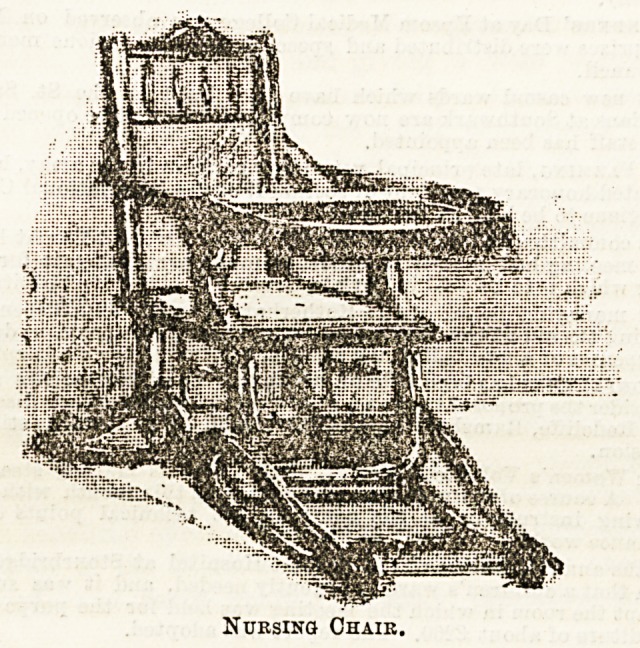# Practical Departments

**Published:** 1894-08-11

**Authors:** 


### The Institutional Workshop.

### PRACTICAL DEPARTMENTS.

### A CHAIR FOR THE NURSERY.

The child's chair here depicted should be recognized as a boon in the nursery, for it may be utilized in several ways according to the requirements of the moment. At will it can be raised in height, by means of the "rockers" upon which it rests, to the level of the table, or, as shown in the drawing,

Nursing Chair.

for use when baby is to be kept quietly out of harm's way, with the small tray for toys, &c.; or again the rockers can be placed in another position and the chair turned into a real rocking chair. The mechanism is simple and easily adjusted. Altogether it is a useful piece of nursery furniture. The manufacturer is Mr. C. Fowle, 85, Victoria Street, S.W.

### ECONOMIC HEATING.

An excellent plan for heating has been followed at the New Laundry Home at Guy's Hospital which was opened some five weeks since. The home itself is so well planned that a description of it must be given later, but the practical advantages of the heating system may have a word of comment to themselves. To begin with, the whole house is heated by pipes. The hot water cisterns are at the top of the house, for the supply of baths, &c., but the two systems are kept entirely separate, as there are two distinct stoves on the ground floor, in the "stoke hole," one of which heats the water supply, the other being only required in winter for the warming of the rooms. These stoves burn coke, except just for lighting, when a little coal is used, and rubbish may be freely burned, thus answering the purpose of destructors as well as heaters. Hot water is laid on all over the house, of course, and there are delightful baths on each floor, with a liberal supply of tip-up wash-hand basins also, all supplied with hot water. The economy of such an arrangement, both as to labour and fuel, may be imagined; indeed, a more excellent could hardly have been planned.

## Figures and Tables

**Figure f1:**